# Genome sequence of *Microvirga lupini* strain LUT6^T^, a novel *Lupinus* alphaproteobacterial microsymbiont from Texas

**DOI:** 10.4056/sigs.5249382

**Published:** 2014-03-01

**Authors:** Wayne Reeve, Matthew Parker, Rui Tian, Lynne Goodwin, Hazuki Teshima, Roxanne Tapia, Cliff Han, James Han, Konstantinos Liolios, Marcel Huntemann, Amrita Pati, Tanja Woyke, Konstantinos Mavromatis, Victor Markowitz, Natalia Ivanova, Nikos Kyrpides

**Affiliations:** 1 Centre for Rhizobium Studies, Murdoch University, Western Australia, Australia; 2 Biological Sciences Department, Binghampton University, New York, USA; 3 Los Alamos National Laboratory, Bioscience Division, Los Alamos, New Mexico, USA; 4 DOE Joint Genome Institute, Walnut Creek, California, USA; 5 Biological Data Management and Technology Center, Lawrence Berkeley National Laboratory, Berkeley, California, USA

**Keywords:** root-nodule bacteria, nitrogen fixation, rhizobia, *Alphaproteobacteria*

## Abstract

*Microvirga lupini* LUT6^T^ is an aerobic, non-motile, Gram-negative, non-spore-forming rod that can exist as a soil saprophyte or as a legume microsymbiont of *Lupinus texensis*. LUT6^T^ was isolated in 2006 from a nodule recovered from the roots of the annual *L. texensis* growing in Travis Co., Texas. LUT6^T^ forms a highly specific nitrogen-fixing symbiosis with endemic *L. texensis* and no other *Lupinus* species can form an effective nitrogen-fixing symbiosis with this isolate. Here we describe the features of *M. lupini* LUT6^T^, together with genome sequence information and its annotation. The 9,633,614 bp improved high quality draft genome is arranged into 160 scaffolds of 1,366 contigs containing 10,864 protein-coding genes and 87 RNA-only encoding genes, and is one of 20 rhizobial genomes sequenced as part of a DOE Joint Genome Institute 2010 Community Sequencing Project.

## Introduction

*Microvirga* is one of the most recently discovered genera of *Proteobacteria* known to engage in symbiotic nitrogen fixation with legume plants, and joins a diverse set of at least twelve other lineages of *Proteobacteria* that share this ecological niche [1-4]. Several genera of legume root-nodule symbionts have a world-wide distribution and interact with many legume taxa. By contrast, symbiotic strains of *Microvirga* are currently known from two distant locations and only two legume host genera [5,6]. The limited geographic and host distribution of *Microvirga* symbionts, along with the fact that root-nodule symbiosis is not characteristic of the genus *Microvirga* as a whole [7], suggest a relatively recent evolutionary transition to legume symbiosis in this group.

*M. lupini* is a specialized nodule symbiont associated with the legume *Lupinus texensis*, an annual plant endemic to a relatively small geographic area in central Texas and northeastern Mexico [5]. The genus *Lupinus* has about 270 annual and perennial species concentrated in western North America and in Andean regions of South America, and a much smaller number of species in the Mediterranean region of Europe and northern Africa [8]. Basal lineages of *Lupinus* all occur in the Mediterranean and are associated with bacterial symbionts in the genus *Bradyrhizobium* [9,10]. *Bradyrhizobium* is also the main symbiont lineage for most *Lupinus* species in North and South America, although a few *Lupinus* species utilize nodule bacteria in the genus *Mesorhizobium* [10-13]. Thus, the acquisition of symbionts in the genus *Microvirga* by plants of *L. texensis* appears to be an unusual, derived condition for this legume genus.

*L. texensis* occurs in grassland and open shrub communities with an annual precipitation of 50 - 100 cm, on diverse soil types [14]. *L. texensis* appears to have a specialized symbiotic relationship with *M. lupini* in that existing surveys have failed to detect nodule symbionts of any other bacterial genus associated with this plant [5]. Moreover, inoculation experiments with other North American species of *Lupinus*, as well as other legume genera, have so far failed to identify any plant besides *L. texensis* that is capable of forming an effective, nitrogen-fixing symbiosis with *M. lupini* [5]. *M. lupini* strain Lut6^T^ was isolated from a nodule collected from a *L. texensis* plant in Travis Co., Texas in 2006. Here we provide an analysis of the complete genome sequence of *M. lupini* strain Lut6^T^; one of the three described symbiotic species of *Microvirga* [15].

## Classification and general features

**Figure 1:**
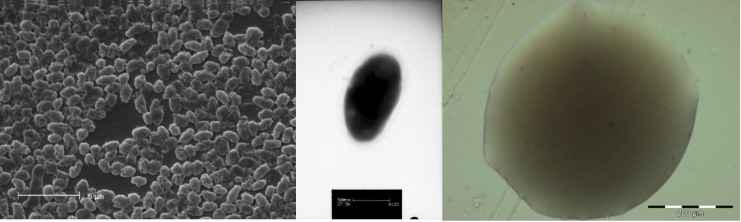
Images of *M. lupini* LUT6^T^ using scanning (Left) and transmission (Center) electron microscopy and the appearance of colony morphology on solid medium (Right).

*M. lupini* LUT6^T^ is a non-motile, Gram-negative rod in the order *Rhizobiales* of the class *Alphaproteobacteria*. The rod-shaped form varies in size with dimensions of 1.0 μm for width and 1.5-2.0 μm for length (Figure 1 Left and Center). It is fast growing, forming colonies within 3-4 days when grown on half strength Lupin Agar (½LA) [16], tryptone-yeast extract agar (TY) [17] or a modified yeast-mannitol agar (YMA) [18] at 28°C. Colonies on ½LA are white-opaque, slightly domed and moderately mucoid with smooth margins (Figure 1 Right).

**Figure 2:**
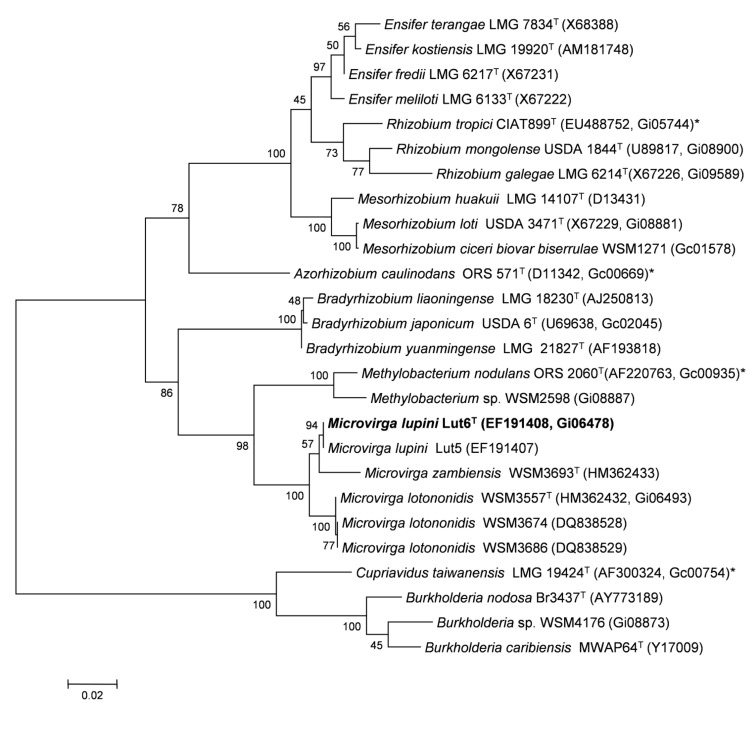
Phylogenetic tree showing the relationship of *M. lupini* LUT6^T^ (shown in bold print) to other root nodule bacteria in the order *Rhizobiales* based on aligned sequences of the 16S rRNA gene (1,320 bp internal region). All sites were informative and there were no gap-containing sites. Phylogenetic analyses were performed using MEGA, version 5 [31]. The tree was built using the Maximum-Likelihood method with the General Time Reversible model [32]. Bootstrap analysis [33] with 500 replicates was performed to assess the support of the clusters. Type strains are indicated with a superscript T. Brackets after the strain name contain a DNA database accession number and/or a GOLD ID (beginning with the prefix G) for a sequencing project registered in GOLD [34]. Published genomes are indicated with an asterisk.

Minimum Information about the Genome Sequence (MIGS) is provided in Table 1. Figure 2 shows the phylogenetic neighbor-hood of *M. lupini* LUT6^T^ in a 16S rRNA sequence based tree. This strain shares 100% (1,358/1,358 bases) and 98% (1,344/1,367 bases) sequence identity to the 16S rRNA of *Microvirga sp.* Lut5 and *Microvirga lotononidis* WSM3557^T^, respectively.

**Table 1:** Classification and general features of *M. lupini* LUT6^T^ according to the MIGS recommendations [19,20]

**MIGS ID**	**Property**	**Term**	**Evidence code**
	Current classification	Domain *Bacteria*	TAS [20]
		Phylum *Proteobacteria*	TAS [21]
		Class *Alphaproteobacteria*	TAS [22,23]
		Order *Rhizobiales*	TAS [23,24]
		Family *Methylobacteriaceae*	TAS [23,25]
		Genus *Microvirga*	TAS [15,26-28]
		Species *Microvirga lupini*	TAS [15]
		Strain LUT6^T^	
	Gram stain	Negative	TAS [15]
	Cell shape	Rod	TAS [15]
	Motility	Non-Motile	IDA
	Sporulation	Non-sporulating	TAS [15]
	Temperature range	Mesophile	TAS [15]
	Optimum temperature	39°C	TAS [15]
	Salinity	Non-halophile	TAS [15]
MIGS-22	Oxygen requirement	Aerobic	TAS [15]
	Carbon source	Varied	TAS [15]
	Energy source	Chemoorganotroph	TAS [15]
MIGS-6	Habitat	Soil, root nodule, on host	TAS [15]
MIGS-15	Biotic relationship	Free living, symbiotic	TAS [15]
MIGS-14	Pathogenicity	Non-pathogenic	NAS
	Biosafety level	1	TAS [29]
	Isolation	Root nodule of *Lupinus texensis*	TAS [5]
MIGS-4	Geographic location	Travis Co., Texas	TAS [5]
MIGS-5	Soil collection date	03 Jan 2006	IDA
MIGS-4.1 MIGS-4.2	Latitude Longitude	-97.838 30.459	IDA IDA
MIGS-4.3	Depth	0-10 cm	IDA
MIGS-4.4	Altitude	270 m	IDA

Evidence codes – IDA: Inferred from Direct Assay; TAS: Traceable Author Statement (i.e., a direct report exists in the literature); NAS: Non-traceable Author Statement (i.e., not directly observed for the living, isolated sample, but based on a generally accepted property for the species, or anecdotal evidence). These evidence codes are from the Gene Ontology project [30].

### Symbiotaxonomy

*M. lupini* strain Lut6^T^ was isolated in from a nodule collected from *Lupinus texensis* growing near Travis Co., Texas. The symbiotic characteristics of this isolate on a range of selected hosts are provided in Table 2.

**Table 2:** Nodulation and N_2_ fixation properties of *M. lupini* Lut6^T^ on selected legumes^†^.

**Legume Species**	**Nodulation**	**N_2_ fixation**	**Comment**
*Lupinus texensis*	Nod^+^	Fix^+^	Highly effective
*Lupinus perennis*	Nod^-^	Fix^-^	No nodulation
*Lupinus succulentus*	Nod^-^	Fix^-^	No nodulation
*Lupinus microcarpus*	Nod^-^	Fix^-^	No nodulation
*Phaseolus vulgaris*	Nod^-^	Fix^-^	No nodulation
*Macroptilium atropurpureum*	Nod^+^	Fix^-^	No fixation
*Desmodium canadense*	Nod^-^	Fix^-^	No nodulation
*Cytisus scoparius*	Nod^+^	Fix^-^	No fixation
*Mimosa pudica*	Nod^-^	Fix^-^	No nodulation

^†^Data compiled [5]. Note that ‘+’ and ‘-’ denote presence or absence, respectively, of nodulation (Nod) or N_2_ fixation (Fix).

## Genome sequencing and annotation information

### Genome project history

This organism was selected for sequencing on the basis of its environmental and agricultural relevance to issues in global carbon cycling, alternative energy production, and biogeochemical importance, and is part of the Community Sequencing Program at the U.S. Department of Energy, Joint Genome Institute (JGI) for projects of relevance to agency missions. The genome project is deposited in the Genomes OnLine Database [34] and an improved-high-quality-draft genome sequence in IMG. Sequencing, finishing and annotation were performed by the JGI. A summary of the project information is shown in Table 3.

**Table 3:** Genome sequencing project information for *M. lupini* LUT6^T^.

**MIGS ID**	**Property**	**Term**
MIGS-31	Finishing quality	Improved high-quality draft
MIGS-28	Libraries used	Illumina GAii shotgun and a paired end 454 libraries
MIGS-29	Sequencing platforms	Illumina GAii and 454 GS FLX Titanium technologies
MIGS-31.2	Sequencing coverage	3.5× 454 paired end, 300× Illumina
MIGS-30	Assemblers	Velvet version 1.0.13; Newbler 2.3, phrap SPS - 4.24
MIGS-32	Gene calling methods	Prodigal 1.4
	GOLD ID	Gi06478
	NCBI project ID	66529
	Database: IMG	2508501050
	Project relevance	Symbiotic N_2_ fixation, agriculture

### Growth conditions and DNA isolation

*M. lupini* LUT6^T^ was cultured to mid logarithmic phase in 60 ml of TY rich media [35] on a gyratory shaker at 28°C. DNA was isolated from the cells using a CTAB (Cetyl trimethyl ammonium bromide) bacterial genomic DNA isolation method [36].

### Genome sequencing and assembly

The genome of *M. lupini* LUT6^T^ was sequenced at the Joint Genome Institute (JGI) using a combination of Illumina [37] and 454 technologies [38]. An Illumina GAii shotgun library which generated 77,090,752 reads totaling 5,858.9 Mbp, and a paired end 454 library with an average insert size of 8 Kbp which generated 238,026 reads totaling 81.4 Mb of 454 data were generated for this genome [36].

All general aspects of library construction and sequencing performed at the JGI can be found at [36]. The initial draft assembly contained 1,719 contigs in 6 scaffolds. The 454 paired end data were assembled together with Newbler, version 2.3-PreRelease-6/30/2009. The Newbler consensus sequences were computationally shredded into 2 Kbp overlapping fake reads (shreds). Illumina sequencing data was assembled with VELVET, version 1.0.13 [39], and the consensus sequence computationally shredded into 1.5 Kbp overlapping fake reads (shreds). The 454 Newbler consensus shreds, the Illumina VELVET consensus shreds and the read pairs in the 454 paired end library were integrated using parallel phrap, version SPS - 4.24 (High Performance Software, LLC). The software Consed [40-42] was used in the following finishing process. Illumina data was used to correct potential base errors and increase consensus quality using the software Polisher developed at JGI [43]. Possible mis-assemblies were corrected using gapResolution (Cliff Han, unpublished) or Dupfinisher [44]. Some gaps between contigs were closed by editing in Consed. The estimated genome size is 10.3 Mb and the final assembly is based on 36.2 Mb of 454 draft data which provides an average 3.5x coverage of the genome and 3,090 Mbp of Illumina draft data which provides an average 300x coverage of the genome.

### Genome annotation

Genes were identified using Prodigal [45] as part of the DOE-JGI annotation pipeline [46]. The predicted CDSs were translated and used to search the National Center for Biotechnology Information (NCBI) nonredundant database, UniProt, TIGRFam, Pfam, PRIAM, KEGG, COG, and InterPro databases. The tRNAScanSE tool [47] was used to find tRNA genes, whereas ribosomal RNA genes were found by searches against models of the ribosomal RNA genes built from SILVA [48]. Other non–coding RNAs such as the RNA components of the protein secretion complex and the RNase P were identified by searching the genome for the corresponding Rfam profiles using INFERNAL [49]. Additional gene prediction analysis and manual functional annotation was performed within the Integrated Microbial Genomes (IMG-ER) platform [50].

## Genome properties

**Figure 3:**
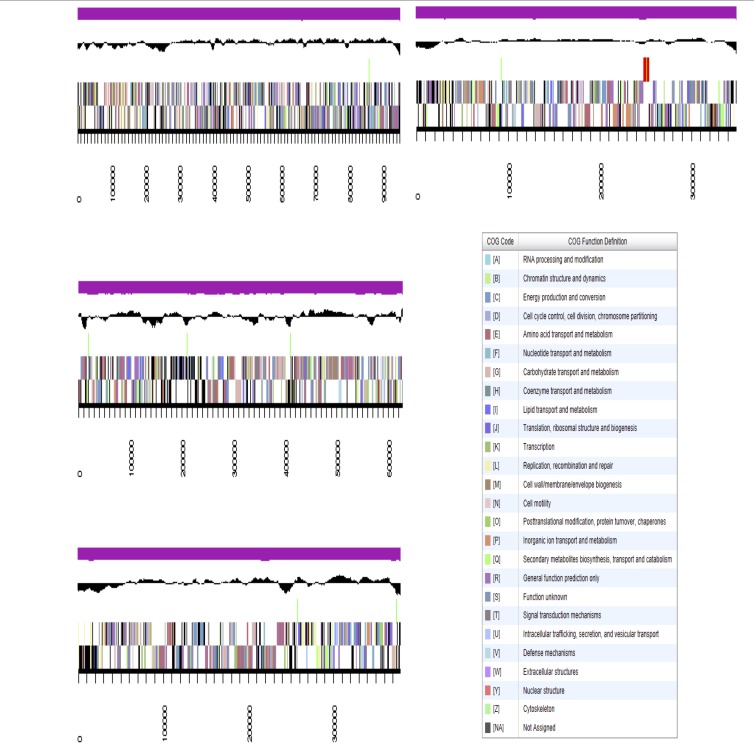
Graphical map of the genome of *Microvirga lupini* LUT6^T^ showing the four largest scaffolds. From bottom to the top of each scaffold: Genes on forward strand (color by COG categories as denoted by the IMG platform), Genes on reverse strand (color by COG categories), RNA genes (tRNAs green, sRNAs red, other RNAs black), GC content, GC skew.

The genome is 9,633,614 nucleotides long with 60.26% GC content (Table 4) and comprised of 160 scaffolds (Figure 3) of 1,366 contigs. From a total of 10,951 genes, 10,864 were protein encoding and 87 RNA only encoding genes. The majority of genes (63.25%) were assigned a putative function whilst the remaining genes were annotated as hypothetical. The distribution of genes into COGs functional categories is presented in Table 5.

**Table 4:** Genome statistics for *Microvirga lupini* LUT6^T^

**Attribute**	**Value**	**% of Total**
Genome size (bp)	9,633,614	100.00
DNA coding region (bp)	7,880,506	81.80
DNA G+C content (bp)	5,805,078	60.26
Number of scaffolds	160	
Number of contigs	1,366	
Total genes	10,951	100.00
RNA genes	87	0.79
rRNA operons	1	0.01
Protein-coding genes	10,864	99.21
Genes with function prediction	6,927	63.25
Genes assigned to COGs	6,990	63.83
Genes assigned Pfam domains	7,343	67.05
Genes with signal peptides	768	7.01
Genes with transmembrane helices	2,006	18.32
CRISPR repeats	0	

**Table 5:** Number of protein coding genes of *Microvirga lupini* LUT6^T^ associated with the general COG functional categories.

**Code**	**Value**	**%age**	**COG Category**
J	209	2.72	Translation, ribosomal structure and biogenesis
A	1	0.01	RNA processing and modification
K	571	7.43	Transcription
L	667	8.68	Replication, recombination and repair
B	10	0.13	Chromatin structure and dynamics
D	53	0.69	Cell cycle control, mitosis and meiosis
Y			Nuclear structure
V	104	1.35	Defense mechanisms
T	463	6.02	Signal transduction mechanisms
M	316	4.11	Cell wall/membrane biogenesis
N	69	0.9	Cell motility
Z	0	0	Cytoskeleton
W	1	0.01	Extracellular structures
U	95	1.24	Intracellular trafficking and secretion
O	249	3.24	Posttranslational modification, protein turnover, chaperones
C	401	5.22	Energy production conversion
G	602	7.83	Carbohydrate transport and metabolism
E	828	10.77	Amino acid transport metabolism
F	100	1.3	Nucleotide transport and metabolism
H	263	3.42	Coenzyme transport and metabolism
I	266	3.46	Lipid transport and metabolism
P	388	5.05	Inorganic ion transport and metabolism
Q	263	3.42	Secondary metabolite biosynthesis, transport and catabolism
R	976	12.70	General function prediction only
S	790	10.28	Function unknown
-	3,961	36.17	Not in COGS

## References

[1] Ardley JK. Symbiotic specificity and nodulation in the southern African legume clade *Lotononis s. l.* and description of novel rhizobial species within the Alphaproteobacterial genus *Microvirga*: Murdoch University, Murdoch, WA, Australia; 2012.

[2] Gyaneshwar P Hirsch AM Moulin L Chen WM Elliott GN Bontemps C Estrada-de Los Santos P Gross E Dos Reis FB Sprent JI Legume-nodulating betaproteobacteria: diversity, host range, and future prospects. Mol Plant Microbe Interact 2011; 24:1276-1288 10.1094/MPMI-06-11-0172 21830951

[3] Maynaud G Willems A Soussou S Vidal C Maure L Moulin L Cleyet-Marel JC Brunel B Molecular and phenotypic characterization of strains nodulating *Anthyllis vulneraria* in mine tailings, and proposal of *Aminobacter anthyllidis* sp. nov., the first definition of *Aminobacter* as legume-nodulating bacteria. Syst Appl Microbiol 2012; 35:65-72 10.1016/j.syapm.2011.11.002 22221859

[4] Willems A The taxonomy of rhizobia; an overview. Plant Soil 2006; 287:3-14 10.1007/s11104-006-9058-7

[5] Andam CP Parker MA Novel alphaproteobacterial root nodule symbiont associated with *Lupinus texensis.* Appl Environ Microbiol 2007; 73:5687-5691 10.1128/AEM.01413-07 17616612 PMC2042092

[6] Yates RJ Howieson JG Reeve WG Nandasena KG Law IJ Bräu L Ardley JK Nistelberger HM Real D O'Hara GW *Lotononis angolensis* forms nitrogen fixing, lupinoid nodules with phylogenetically unique, fast-growing, pink-pigmented bacteria, which do not nodulate *L. bainesii* or *L. listii.* Soil Biol Biochem 2007; 39:1680-1688 10.1016/j.soilbio.2007.01.025

[7] Weon HY Kwon SW Son JA Jo EH Kim SJ Kim YS Kim BY Ka JO Description of *Microvirga aerophila* sp. nov. and *Microvirga aerilata* sp. nov., isolated from air, reclassification of *Balneimonas flocculans* Takeda et al. 2004 as *Microvirga flocculans* comb. nov. and emended description of the genus *Microvirga.* Int J Syst Evol Microbiol 2010; 60:2596-2600 10.1099/ijs.0.018770-0 20023055

[8] Drummond CS Eastwood RJ Miotto ST Hughes CE Multiple continental radiations and correlates of diversification in *Lupinus* (Leguminosae): testing for key innovation with incomplete taxon sampling. Syst Biol 2012; 61:443-460 10.1093/sysbio/syr126 22228799 PMC3329764

[9] Jarabo-Lorenzo A Velazquez E Perez-Galdona R Vega-Hernandez MC Martinez-Molina E Mateos PF Vinuesa P Martinez-Romero E Leon-Barrios M Restriction fragment length polymorphism analysis of 16S rDNA and low molecular weight RNA profiling of rhizobial isolates from shrubby legumes endemic to the Canary islands. Syst Appl Microbiol 2000; 23:418-425 10.1016/S0723-2020(00)80073-9 11108022

[10] Stepkowski T Hughes CE Law IJ Markiewicz L Gurda D Chlebicka A Moulin L Diversification of lupine *Bradyrhizobium* strains: evidence from nodulation gene trees. Appl Environ Microbiol 2007; 73:3254-3264 10.1128/AEM.02125-06 17400786 PMC1907101

[11] Barrera LL Trujillo ME Goodfellow M Garcia FJ Hernandez-Lucas I Davila G van Berkum P Martinez-Romero E Biodiversity of bradyrhizobia nodulating *Lupinus* spp. Int J Syst Evol Microbiol 1997; 47:1086-1091 9336911 10.1099/00207713-47-4-1086

[12] Koppell JH Parker MA Phylogenetic clustering of *Bradyrhizobium* symbionts on legumes indigenous to North America. Microbiology 2012; 158:2050-2059 10.1099/mic.0.059238-0 22539163

[13] Simms EL, Taylor DL, Povich J, Shefferson RP, Sachs JL, Urbina M, Tausczik Y. An empirical test of partner choice mechanisms in a wild legume-*Rhizobium* interaction. Proceedings of Biological Sciences 2006;273(1582):77-81. 10.1098/rspb.2005.3292 PMC1560009 16519238

[14] Nixon ES Edaphic responses of *Lupinus texensis* and *Lupinus subcarnosus.* Ecology 1964; 45:459-469 10.2307/1936099

[15] Ardley JK Parker MA De Meyer SE Trengove RD O'Hara GW Reeve WG Yates RJ Dilworth MJ Willems A Howieson JG *Microvirga lupini* sp. nov., *Microvirga lotononidis* sp. nov. and *Microvirga zambiensis* sp. nov. are alphaproteobacterial root-nodule bacteria that specifically nodulate and fix nitrogen with geographically and taxonomically separate legume hosts. Int J Syst Evol Microbiol 2012; 62:2579-2588 10.1099/ijs.0.035097-0 22199210

[16] Howieson JG Ewing MA D'antuono MF Selection for acid tolerance in *Rhizobium meliloti.* Plant Soil 1988; 105:179-188 10.1007/BF02376781

[17] Beringer JE R factor transfer in *Rhizobium leguminosarum.* J Gen Microbiol 1974; 84:188-198 10.1099/00221287-84-1-188 4612098

[18] Terpolilli JJ. Why are the symbioses between some genotypes of *Sinorhizobium* and *Medicago* suboptimal for N_2_ fixation? Perth: Murdoch University; 2009. 223 p.

[19] Field D Garrity G Gray T Morrison N Selengut J Sterk P Tatusova T Thomson N Allen M Angiuoli SV Towards a richer description of our complete collection of genomes and metagenomes "Minimum Information about a Genome Sequence " (MIGS) specification. Nat Biotechnol 2008; 26:541-547 10.1038/nbt1360 18464787 PMC2409278

[20] Woese CR Kandler O Wheelis ML Towards a natural system of organisms: proposal for the domains *Archaea, Bacteria*, and *Eucarya.* Proc Natl Acad Sci USA 1990; 87:4576-4579 10.1073/pnas.87.12.4576 2112744 PMC54159

[21] Garrity GM, Bell JA, Lilburn T. Phylum XIV. *Proteobacteria* phyl. nov. In: Garrity GM, Brenner DJ, Krieg NR, Staley JT (eds), Bergey's Manual of Systematic Bacteriology, Second Edition, Volume 2, Part B, Springer, New York, 2005, p. 1

[22] Garrity GM, Bell JA, Lilburn T. Class I. *Alphaproteobacteria* class. nov. In: Garrity GM, Brenner DJ, Krieg NR, Staley JT (eds), Bergey's Manual of Systematic Bacteriology, Second Edition, Volume 2, Part C, Springer, New York, 2005, p. 1.

[23] Validation List No. 107. List of new names and new combinations previously effectively, but not validly, published. Int J Syst Evol Microbiol 2006; 56:1-6 10.1099/ijs.0.64188-0 16403855

[24] Kuykendall LD. Order VI. *Rhizobiales* ord. nov. In: Garrity GM, Brenner DJ, Kreig NR, Staley JT, editors. Bergy's Manual of Systematic Bacteriology. Second ed: New York: Springer - Verlag; 2005. p 324.

[25] Garrity GM, Bell JA, Lilburn T. Family IX. *Methylobacteriaceae* fam. nov. In: Garrity GM, Brenner DJ, Krieg NR, Staley JT (eds), Bergey's Manual of Systematic Bacteriology, Second Edition, Volume 2, Part C, Springer, New York, 2005, p. 567.

[26] Kanso S Patel BKC *Microvirga subterranea* gen. nov., sp. nov., a moderate thermophile from a deep subsurface Australian thermal aquifer. Int J Syst Evol Microbiol 2003; 53:401-406 10.1099/ijs.0.02348-0 12710604

[27] Zhang J Song F Xin YH Zhang J Fang C *Microvirga guangxiensis* sp. nov., a novel alphaproteobacterium from soil, and emended description of the genus Microvirga. Int J Syst Evol Microbiol 2009; 59:1997-2001 10.1099/ijs.0.007997-0 19567564

[28] Weon H-Y Kwon S-W Son J-A Jo E-H Kim S-J Kim Y-S Kim B-Y Ka J-O Description of *Microvirga aerophila* sp. nov. and *Microvirga aerilata* sp. nov., isolated from air, reclassification of *Balneimonas flocculans* Takeda et al. 2004 as Microvirga flocculans comb. nov. and emended description of the genus Microvirga. Int J Syst Evol Microbiol 2010; 60:2596-2600 10.1099/ijs.0.018770-0 20023055

[29] Gubler M Hennecke H Fix A. B and C genes are essential for symbiotic and free-living, microaerobic nitrogen fixation. FEBS Lett 1986; 200:186-192 10.1016/0014-5793(86)80536-1

[30] Ashburner M Ball CA Blake JA Botstein D Butler H Cherry JM Davis AP Dolinski K Dwight SS Eppig JT Gene ontology: tool for the unification of biology. The Gene Ontology Consortium. Nat Genet 2000; 25:25-29 10.1038/75556 10802651 PMC3037419

[31] Tamura K Peterson D Peterson N Stecher G Nei M Kumar S MEGA5: Molecular Evolutionary Genetics Analysis using Maximum Likelihood, Evolutionary Distance, and Maximum Parsimony Methods. Mol Biol Evol 2011; 28:2731-2739 10.1093/molbev/msr121 21546353 PMC3203626

[32] Nei M, Kumar S. Molecular Evolution and Phylogenetics. New York: Oxford University Press; 2000.

[33] Felsenstein J Confidence limits on phylogenies: an approach using the bootstrap. Evolution 1985; 39:783-791 10.2307/2408678 28561359

[34] Liolios K Mavromatis K Tavernarakis N Kyrpides NC The Genomes On Line Database (GOLD) in 2007: status of genomic and metagenomic projects and their associated metadata. Nucleic Acids Res 2008; 36:D475-D479 10.1093/nar/gkm884 17981842 PMC2238992

[35] Reeve WG Tiwari RP Worsley PS Dilworth MJ Glenn AR Howieson JG Constructs for insertional mutagenesis, transcriptional signal localization and gene regulation studies in root nodule and other bacteria. Microbiology 1999; 145:1307-1316 10.1099/13500872-145-6-1307 10411257

[36] DOE Joint Genome Institute user homepage. http://my.jgi.doe.gov/general/index.html

[37] Bennett S Solexa Ltd. Pharmacogenomics 2004; 5:433-438 10.1517/14622416.5.4.433 15165179

[38] Margulies M Egholm M Altman WE Attiya S Bader JS Bemben LA Berka J Braverman MS Chen YJ Chen Z Genome sequencing in microfabricated high-density picolitre reactors. Nature 2005; 437:376-380 16056220 10.1038/nature03959 PMC1464427

[39] Zerbino DR. Using the Velvet *de novo* assembler for short-read sequencing technologies. Current Protocols in Bioinformatics 2010;Chapter 11:Unit 11 5. 10.1002/0471250953.bi1105s31 PMC2952100 20836074

[40] Ewing B Green P Base-calling of automated sequencer traces using phred. II. Error probabilities. Genome Res 1998; 8:186-194 10.1101/gr.8.3.175 9521922

[41] Ewing B Hillier L Wendl MC Green P Base-calling of automated sequencer traces using phred. I. Accuracy assessment. Genome Res 1998; 8:175-185 10.1101/gr.8.3.175 9521921

[42] Gordon D Abajian C Green P Consed: a graphical tool for sequence finishing. Genome Res 1998; 8:195-202 10.1101/gr.8.3.195 9521923

[43] LaButti K, Foster B, Lowry S, Trong S, Goltsman E, Lapidus A. POLISHER: a Tool for Using Ultra Short Read in Microbial Genome Finishing http://publications.lbl.gov/fedora/repository/ir%3A150163 Berkeley Lab Publications 2008.

[44] Han C, Chain P. Finishing repeat regions automatically with Dupfinisher. In: Valafar HRAH, editor. Proceeding of the 2006 international conference on bioinformatics & computational biology: CSREA Press; 2006. p 141-146.

[45] Hyatt D Chen GL Locascio PF Land ML Larimer FW Hauser LJ Prodigal: prokaryotic gene recognition and translation initiation site identification. BMC Bioinformatics 2010; 11:119 10.1186/1471-2105-11-119 20211023 PMC2848648

[46] Mavromatis K Ivanova NN Chen IM Szeto E Markowitz VM Kyrpides NC The DOE-JGI Standard operating procedure for the annotations of microbial genomes. Stand Genomic Sci 2009; 1:63-67 10.4056/sigs.632 21304638 PMC3035208

[47] Lowe TM Eddy SR tRNAscan-SE: a program for improved detection of transfer RNA genes in genomic sequence. Nucleic Acids Res 1997; 25:955-964 10.1093/nar/25.5.0955 9023104 PMC146525

[48] Pruesse E Quast C Knittel K Fuchs BdM, Ludwig W, Peplies J, Glöckner FO. SILVA: a comprehensive online resource for quality checked and aligned ribosomal RNA sequence data compatible with ARB. Nucleic Acids Res 2007; 35:7188-7196 10.1093/nar/gkm864 17947321 PMC2175337

[49] INFERNAL http://infernal.janelia.org

[50] Markowitz VM Mavromatis K Ivanova NN Chen IM Chu K Kyrpides NC IMG ER: a system for microbial genome annotation expert review and curation. Bioinformatics 2009; 25:2271-2278 10.1093/bioinformatics/btp393 19561336

